# Correlates to the variable effects of cannabis in young adults: a preliminary study

**DOI:** 10.1186/1477-7517-9-15

**Published:** 2012-03-30

**Authors:** Ariella A Camera, Veronica Tomaselli, Jerry Fleming, Gul A Jabbar, Melissa Trachtenberg, Juan A Galvez-Buccollini, Ashley C Proal, Richard N Rosenthal, Lynn E DeLisi

**Affiliations:** 1 VA Boston Healthcare System, 940 Belmont Street, Brockton, MA, 02301, USA; 2 St. Luke’s Roosevelt Hospital Center, 1111 Amsterdam Avenue, New York, NY, 10027, USA; 3 Harvard Medical School, 25 Shattack Street, Boston, MA, 02115, USA; 4 Langone School of Medicine, New York University, 550 First Avenue, New York, NY, 10016, USA

## Abstract

**Background:**

Cannabis use can frequently have adverse affects in those that use it and these can be amplified by various characteristics of an individual, from demographic and environmental variations to familial predisposition for mental illnesses.

**Methods:**

The current study of 100 individuals, who were cannabis users during their adolescence and may still be users, was a survey of the self perceived effects of cannabis and their correlates. A reliable family member was also interviewed for determination of family history of various major mental illnesses and substance use.

**Results:**

As many as 40% of cannabis users had paranoid feelings (suspiciousness) when using cannabis, although the most frequent effect was feeling relaxed (46%). Having a familial background for mental illnesses such as depression or schizophrenia did not determine the effects of cannabis nor its pattern of use, although the number of subjects with such a history was small. An age at which an individual began using cannabis did have an effect on how heavily it was used and the heavier the cannabis use, the more likely the individual was also to have had psychotic symptoms after use. There were no sex differences in effects of cannabis. These results are tempered by the reliance on self-report for many of the variables ascertained.

**Conclusion:**

Cannabis can frequently have negative effects in its users, which can be amplified by certain demographic and/or psychosocial factors. Thus, users with a specific profile may be at a higher risk of unpleasant effects from cannabis use and caution should be noted when cannabis is administered to young people for medicinal purposes.

## Background

Cannabis use is wide-spread among adolescents and young adults in the USA and world-wide [1]. There has also been considerable debate about whether it is truly a dangerous recreational drug or is relatively harmless and should be legalized [2]. The outcome of this debate depends largely on whether cannabis can be shown to cause deleterious changes in the brain and cognition. While some changes in memory have been noted in habitual cannabis users, [3,4] they may recover after cessation of drug use [5] and there has never been consistently replicated evidence of clear structural or structural brain changes in heavy users [6].

Quantitative analysis on cannabis users and its effects presents researchers with several challenges. First, it is difficult to quantify the precise amounts of the drug that are taken by each user over time given that the quantity varies considerably depending on the quality of purchased drug, and the terminology used. The majority of cannabis users describe their use in terms of “joints”, “bowls” or “blunts” which can all vary in volume [7,8]. Moreover, those individuals who have used cannabis heavily have likely tried and been frequent users of other drugs as well. At a minimum they seem to be frequent alcohol users. Thus it is difficult to tease apart the effects of cannabis from other substances.

Why young people have the need to use cannabis on a frequent basis is an interesting question and may be associated with their underlying personality characteristics, environment during childhood and adolescence, as well as any psychiatric disturbances they have experienced, such as depression, anxiety or even psychotic symptoms [1]. Family history of these disorders may also influence characteristics of cannabis use, as symptoms in genetically vulnerable populations could be initiated by heavy substance use.

When people use cannabis, it may acutely produce a wide variety of effects, from a feeling of euphoria and well being, to one of anxiety and irritability. Why the effects vary so much between individuals is unknown, but is likely to be associated with genetic, environmental, age, gender and other psychological factors as listed above. In the current study we aimed to explore this question further.

## Methods

### Sample

One hundred individuals from the New York City community participated in this study. They were recruited by advertizing in the electronic classified program, “Craig’s List” requesting research volunteers between the ages of 18–35 who frequently use or used cannabis initially during their adolescent years. Potential study participants were telephone screened and eliminated if found to meet the following exclusion criteria: previous hospitalization for a psychiatric illness, admitted to using other recreational drugs other than cannabis more than 5 times in a lifetime, did not have a close family informant available for obtaining family history information. Cannabis use during adolescence was required because this period of life is postulated to be a time in brain development that could be more vulnerable to the effects of cannabis than later in adulthood.

### Measures

All 100 study participants were interviewed using the Diagnostic Interview for Genetic Studies [9] supplemented by questions taken from the Psychiatric Research Interview for Substance and Mental Disorders [10] and combined with the Structured Interview for Schizotypy [11]. A family pedigree was drawn with information obtained from the participant, and when available, at least one family informant was interviewed regarding illnesses known to occur within the family using the Family Interview for Genetic Studies [12], a structured interview aimed at obtaining information about family members from a reliable and knowledgeable family informant. All individuals and their family members participating in this study received code numbers and no identifying information was obtained about other ill members in the family. This protocol was approved by the Institutional Review Boards of: The New York University Langone School of Medicine; St. Luke’s Roosevelt Hospital Center, New York City; The Nathan Kline Institute, Orangeburg, NY; and the VA Boston Healthcare System where Dr. DeLisi was employed.

The effects felt by each participant while administering cannabis were solicited by a structured oral questionnaire aimed at yes/no answers about a variety of specific experiences (see Table 1).

**Table 1:** Self reported effects of cannabis acutely

**Controls N = 100**		
**Variable #**	**Variable description**	**Reported yes (%)**
1	Paranoia, Delusions	40
2	Hallucinations	7
3	Relaxed, Calm, Clear Minded	46
4	Altered Appetite	39
5	Altered Sleeping Patterns, Tired, Sleepy	17
6	Disturbed, Bad Highs, Disruption of Daily Activities	7
7	Sad, Lonely, Depressed, Lack of Motivation, Lazy	12
8	Altered Concentration, Loss of Memory	25
9	Anxious, Irritable, Panic Attacks	11
10	Altered Sex Drive	11
11	Heightened Senses, Perception, Intense Feelings, Mood Swings, Alertness	11
12	Fun, Happy, Euphoric, Better, More Enjoyable	20
13	Headache, Light Headed, Feint, high Blood Pressure	10
14	Racing Thoughts, Fixated Thoughts	7
15	Introspective, Insightful, Creative	7
16	Loss of Reality, Feeling Weird, Body Sensations, Out of It	15

All coded data extracted from interviews including demographic information, psychiatric symptoms, diagnoses, alcohol use, amount of cannabis use, age of onset, and acute effects of cannabis were entered into an Excel database by one researcher and data rechecked for accuracy. DSM-IV diagnoses (Axis I for major psychiatric illness and Axis II for personality disorders) were made on all participants by a research psychiatrist using all available information collected during the interview.

### Statistical analysis

All statistical analyses were performed using SPSS, version 18. Univariate analyses were used to examine the frequency distribution across individual variables and are presented in the paper as the descriptive analysis. For univariate analyses of continuous variables, means and standard deviations were calculated. A number of bivariate analyses (simultaneous analysis of two variables) were performed to see if one variable is related to another variable. For comparison of nominal (categorical) bivariate data, the chi-square statistic was used (i.e. Race by Gender). For comparison of interval (continuous) bivariate data the independent t-test statistic was used (i.e. age at onset in males vs. females). Other associations and causal relationships were looked at by calculating correlation coefficients. The Pearson’s correlation was used to find a correlation between at least two continuous variables. In the case of analyzing the correlation between two binary variables (i.e. yes-no, absent-present) we used the Phi-statistic. The phi coefficient is a measure of the degree of association between two binary variables and is similar to the correlation coefficient in its interpretation. Since we examined a number of pair-wise comparisons, a conservative Bonferroni adjustment was used to manage multiple significant levels (p-values).

For estimating the intensity (quantity-frequency) of cannabis use, a Likert scale was created to assign numbers from 1 to 5 based on the frequency of cannabis use by the subjects. The scale consisted of ranging from 5 = very frequent, 4 = frequent, 3 = occasionally, 2 = rarely, and 1 = very rarely. For example, heavy Cannabis users who used multiple times a day were assigned a 5 and subjects that used cannabis 1–2 times a month were assigned a 2. Next, independent t-tests were used to compare the frequency of usage with the cannabis self reported symptoms. Pearson correlations were used to correlate cannabis frequency with other quantitative variables such as age of onset of Cannabis use and duration of use.

A factor analysis was applied to the data set in order to determine whether the list of 16 cannabis induced effect symptoms might be attributed largely or entirely by a much smaller number of variables. Factor analysis is often used to identify a small number of factors that explain most of the variance observed in a much larger number of manifest variables. The assumption here is that underneath our indicators there are continuous latent variables which allow for factor analysis and the corresponding correlation matrix. We used Principal Component Analysis as the extraction method employing Varimax Rotation. Components extracted consisted of variables with a factor loading score of .40 or greater. Each factor was validated by examining the corresponding Pearson and Phi correlation matrix to determine that the defining variables were highly correlated. Once the factors have been determined SPSS creates one new variable for each factor in the final solution. These new factor variables can in-turn by used in further analyses of association with other original variables.

## Results

Table 2 lists all demographic characteristics of the cohort. The study included 48 males (mean age +/−SD: 22.54 +/− 2.60) and 52 females (21.83+/−2.63). 75% of the individuals had no psychiatric diagnoses, while 19% were diagnosed with major depression, 9% satisfied criteria for schizotypal personality disorder, 4% for paranoid personality disorder, and 9% for other personality disorders or traits partially satisfying criteria for a spectrum of personality disorders. 26% individuals scored positively on the SIS for social anxiety.

**Table 2:** Demographic variables of the cohort

	**Controls N = 100**	**Males N = 48**	**Females N = 52**
***Demographics***			
**Age**			
Mean (SD)	22.17 (2.63)	22.54 (2.60)	21.83 (2.63)
**Age Depression Diagnosis**			
Mean (SD)	17.54 (3.64)	17.43 (4.2)	17.67 (3.27)
**Age Onset of Alcohol Usage**			
Mean (SD)	15.91 (2.38)	15.73 (2.54)	16.09 (2.23)
**Race/Ethnicity (%)**			
Caucasian	40 (40%)	25 (52%)	15 (29%)
African American, Black	27 (27%)	13 (27%)	14 (27%)
Hispanic, Latino	24 (24%)	7 (15%)	17 (33%)
Asian	4 (4%)	1 (2%)	3 (6%)
Mixed	5 (5%)	2 (4%)	3 (6%)
**Psychiatric Lifetime Diagnoses**			
No Diagnosis	75%	35 (73%)	40 (77%)
Depression	19%	9 (19%)	10 (19%)
Schizotypal Traits	9%	3 (6%)	6 (12%)
Paranoid Personality	4%	2 (4%)	2 (4%)
Antisocial Personality	5%	5 (10%)	0
Social Anxiety	26%	12 (25%)	14 (27%)
ADHD	2%	2 (4%)	0
Dysthymia	2%	1 (2%)	1(2%)
**Alcohol Use**	91%	45(94%)	46 ( 89%
**Family History**			
Schizophrenia	10%	3(6%)	7 (14%)
Manic Depression	14%	8 (17%)	6 (12%)
Depression	33%	15 (31%)	18 (35%)
Alcohol Abuse	32%	15 (31%)	17(33%)
Drug Abuse	35%	16(33%)	19 (37%)

The variety of self-reported effects of cannabis was reduced to 16 major types and the proportion of subjects having them listed in Table 1. The most common effect was to feel relaxed, calm and clear minded (46%), while as many as 40% experienced suspiciousness (labeled as feeling “paranoid” and other delusions). 39% of the controls reported to have an altered appetite and 25% admitted to altered concentration and some memory loss.

Factor analyses of all cannabis effects in Table 1 using a Rotated Component Matrix resulted in 5 main components listed in Table 3: Factor 1(increased irritability/intensity); Factor 2 (relaxed); Factor 3 (Hallucinations/delusions); Factor 4: (Hallucinations and other perceptual disturbances); Factor 5: (depression).

**Table 3:** **Factor analyses of all effects in table**6**(rotated component matrix)**

**Variable #**	**Variable description**	**Component**				
		**1**	**2**	**3**	**4**	**5**
1	Paranoia, Delusions			0.58		
2	Hallucinations			0.43	0.49	
3	Relaxed, Calm, Clear Minded		0.80			
4	Altered Appetite		0.75			
5	Altered Sleeping Patterns, Tired, Sleepy					0.75
6	Disturbed, Bad Highs, Disruption of Daily Activities				0.71	
7	Sad, Lonely, Depressed, Lack of Motivation, Lazy					0.80
8	Altered Concentration, Loss of Memory					
9	Anxious, Irritable, Panic Attacks	0.55				
10	Altered Sex Drive					
11	Heightened Senses, Perception, Intense Feelings, Mood Swings, Alertness	0.72				
12	Fun, Happy, Euphoric, Better, More Enjoyable	0.50				
13	Headache, Light Headed, Faint, High B.P.			0.81		
14	Racing Thoughts, Fixated Thoughts					
15	Introspective, Insightful, Creative	0.70				
16	Loss of Reality, Feeling Weird, Body Sensations, Out of It				0.72	
Extraction Method: Principal Component Analysis						
Rotation Method: Varimax with Kaiser Normalization						
a. Rotation converged in 13 iterations						

Note that 5 main components emerge: Factor 1(increased irritability/intensity); Factor 2 (relaxed); Factor 3 (Hallucinations/delusions); Factor 4 (Hallucinations and other perceptual disturbances); Factor 5 (depression).

In order to determine variables that might be associated with main components of the effects of cannabis, both T tests and Pearson’s correlations were performed reflected in Table 4. Both factors 2 and 4 were associated with having had at least 1 episode of major depression (T = 4.3, p <0.0000 and T = 2.36, p = 0.02). Referring to Table 5, having a family history of schizophrenia in a first or second degree relative was associated with factor 1 (T = 2.15, p = .04). Factor 3 was associated with age of onset of cannabis use (Pearson’s r = −0.29, p <0.004) so that the earlier the onset of use the greater the likelihood of feeling paranoid (suspiciousness) and having other delusions with use. The age of onset of alcohol use was significantly correlated with the age of onset of cannabis use (r = .28, p <0.01).

**Table 4:** Significant correlations of cannabis use

	**Correlation**	
**Variables**	**Coefficient**	**P value**
Age & Duration of MJ usage	0.582	0.000
Age & Age Diagnosis Depression	0.581	0.037
Age of Onset MJ Use & Duration of MJ Use	−0.517	0.000
Age of onset Alcohol Use & Age of onset MJ Use	0.450	0.000
Age of onset MJ use & Factor Component 3 (Paranoia/Delusions & Hallucinations)	−0.255	0.010
MJ Frequency of use &Years of MJ Use	0.318	0.001
MJ Frequency of use & Age of onset MJ Use	−0.301	0.002
Age^b^ onset of MJ use & Altered concentration and memory	0.222*	0.027

a. The first seven correlations are Pearson, 24 analyses ran and the eighth correlation is PHI, 16 analyses ran.

b. Based on the two categories above and below the mean.

c. Bonferonni Correction not used regarding these correlations.

**Table 5:** Factor scores & variables

**Variables**	**N**	**Mean**	**T Value**	**P value**
**Age MJ Use**^**a**^		Factor Score 3		
Absent	43	0.329	2.963	0.004
Present	57	−0.248		
**Depression**		Factor Score 2		
Absent	81	0.154	4.28	0
Present	19	−0.657		
**Depression**		Factor Score 4		
Absent	81	0.073	2.362	0.021
Present	19	−0.313		
**Social Anxiety**		Factor Score 2		
Absent	74	1.02	2.891	0.005
Present	26	0.8		
**Fam Hx Schizo**		Factor Score 1		
Absent	90	0.039	2.151	0.043
Present	10	−0.351		
**Fam Hx MD**		Factor Score 5		
Absent	86	0.9	−2.089	0.039
Present	14	1.41		
**Family Hx Alc**		Factor Score 2		
Absent	68	0.137	2.036	0.046
Present	32	−0.291		
**Family Hx Alc**		Factor Score 3		
Absent	68	−0.148	−2.204	0.03
Present	32	0.315		

a. Based on the two categories above and below the mean, 16 years old.

b. Variables: Hx = History, BP = Bipolar Disorder, Alc = Alcoholism.

Chi-square analyses revealed that those cannabis users who also used alcohol were significantly more likely to report having hallucinations with cannabis use (chi-square = 10.5, p <0.001). In Table 6, those cannabis users who experienced at least one episode of depression were significantly more likely to feel relaxed, calm and clear minded with cannabis use (chi-square = 5.9, p <0.01), as well as having reported an altered appetite (chi-square = 11.2, p <0.001). Having a family history of bipolar disorder was significantly related to feeling depressed, sad, lonely with lack of motivation with cannabis use (chi-square = 8.7, p <0.003). Having a family history of alcohol abuse was significantly related to having racing and fixated thoughts with cannabis use (chi-square =10.0, p <0.002). Family history of alcoholism was not associated with alcohol usage of the cannabis users (chi-square = 2.5, p <0.112). Also family history of Bipolar Disorder was not associated with reporting feeling sad, lonely, or depressed while using cannabis (chi-square = 8.7, p <0.003), (See Table 7).

**Table 6:** Significant chi-square analyses for symptoms of cannabis

	**Cannabis symptoms**		**CHI**	**P-value**
	Hallucinations			
**Alcohol Usage**	No	Yes		
Absent	6	87	10.5	0.001
Present	3	4		
	Alt. Appetite			
**Social Anxiety**	No	Yes		
Absent	39	35	8.236	0.004
Present	22	4		
	Relax/Calm/Clear			
**Depression**	No	Yes		
Absent	39	42	5.877	0.015
Present	15	4		
	Alt. Appetite			
**Depression**	No	Yes		0.001
Absent	43	38	11.22	
Present	18	1		

a. Variables: Hx = History, BP = Bipolar Disorder, Alc = Alcoholism.

**Table 7:** Family history of major depression (MD), alcoholism (Alc),schizophrenia (Schiz) or bipolar disorder (BP) and cannabis symptoms

**Family Hx**	**Symptoms**		**CHI**	**P-value**
	Sad, Lonely etc.			
**Family Hx MD**	No	Yes		
Absent	79	9	8.7	0.003
Present	7	5		
	Racing thoughts etc.			
**Family Hx Alc**	No	Yes		
Absent	67	26	10	0.002
Present	1	6		
	Alcohol Usage			
**Family Hx Alc**	No	Yes		
Absent	4	64	2.5	0.112
Present	5	27		
	Hallucinations			
**Family Hx Schiz**	No	Yes		
Absent	84	6	0.2	0.695
Present	9	1		
	Sad Lonely etc.			
**Family Hx BP**	No	Yes		
Absent	79	9	8.7	0.003
Present	7	5		

a. Variables: Hx = History, BP = Bipolar Disorder, Alc = Alcoholism.

All other analyses were non significant, except having a family history of bipolar disorder which was associated with depressive symptoms (chi-square = 5.206, p = 0.023). Having a family history of schizophrenia was not associated with having delusions or hallucinations (chi-square = 0.154; p = 0.695), although the N for those with a family history was small and may be underpowered (10/100), as a 95% CI around the observed odds ratio ranges between 0.17 and 14.44. Having a past history of major depression was not associated with feeling depressed after cannabis use (factor 5). Males and females did not appear to have any different effects of cannabis.

## Discussion

Cannabis use produces variable effects in different users. Because cannabis can be considered a major public health problem when used frequently, and yet has now been legalized in some places for medicinal purposes, it would be important to know what characteristics of individuals make them more vulnerable to adverse subjective effects of cannabis, the so-called “bad trips.” Family history of psychiatric illnesses was ascertained from family member interviews in order to determine if a genetic predisposition could determine the types of effects one gets when using cannabis. Since major depression is a biologically-based disorder with a genetic predisposition, any correlations with a history of depression may make one more biologically vulnerable to becoming depressed when using cannabis. However, having been prone to major depression did not appear to be associated with becoming depressed with cannabis use, while having a family history of bipolar disorder did. Interestingly, a history of past major depression was associated with not only the “relaxed” factor, but also with hallucinations and perceptual alternations. Although having a family history of schizophrenia was associated with increased irritability and intensity, it was not associated with hallucinations or having paranoia and other delusions. While interesting, our failure to find this association may be due to the limited power of this study (having only 10 probands with a family history of schizophrenia). Sex or ethnicity of the individual did not appear to determine what types of effects of cannabis were reported. An explanation for these associations or lack of is not clear and will require replication in larger studies.

The earlier the age of onset of cannabis use, the more likely one experienced delusions or paranoia. One might speculate that this effect may have to do with the level of brain maturation at the time of onset of use and how much cannabis can then alter the course of that development.

Twenty-six percent of individuals in this study met lifetime criteria for social anxiety disorder, a similar association having been reported by [13-15]. Perhaps this personality trait may lead people to use cannabis so that they can feel more relaxed in social situations, although we do not have an estimate of the percentage of non-cannabis using persons who also exhibit social anxiety and this may simply be a common trait in general.

Barkus and Lewis [16] reported that schizotypy was associated with psychosis-like effects of cannabis and proposed that people with schizotypy might be more vulnerable to developing a psychosis when using cannabis. The current study fails to confirm this finding. People with schizotypal personality disorder or any schizotypal traits did not more frequently report any evidence of psychosis than those who did not have these traits.

Limitations of this study include (1) many of the variables ascertained were provided by self-report by each subject studied and thus relying on not only retrospective recall of events, but truthfulness of each individual, which is an important drawback of this study. Only a prospective longitudinal study with objective measures observed by a third party would be more definitive. And (2), that our numbers for those individuals whose family members reported major mental illness among relatives were small and thus our negative findings are not definitive and need to be confirmed in larger studies.

In summary, we were unable to show that having a family history of a psychotic illness and thus presumably carrying genes for these illnesses could lead to a tendency to have symptoms characteristic of these illnesses when using cannabis. This could simply be due to the small N of those with family history of mental illness, or could be an initial important finding, given that there is much controversy about whether cannabis itself can cause schizophrenia and has long been considered an environmental risk factor for developing this illness [17]. Larger studies will need to be performed with greater power to see if this finding is confirmed. Nevertheless, it is notable that there are many different effects of cannabis when used as a recreational drug and only 20% of the participants in this study felt “euphoric and happy” with its use, while 40% reported feeling paranoid. These variable effects of the drug and their frequency should be considered as cannabis now has become legalized in several states in the USA and world-wide for a wide variety of medicinal purposes.

## Competing interests

The authors declare that they have no competing interests.

## Authors’ contributions

AAC coordinated data files and completed all data analyses. VT coordinated research activities at all New York research sites. JF consulted and assisted in statistical analyses. GAJ assisted in data collection. MT assisted in conducting clinical interviews and coordinating research project in New York. JAG-B assisted with data collection and analyses. RNR contributed to the project design. LED developed and directed all aspects of research project. All authors read and approved the final manuscript.
